# Cyberknife, Helical Tomotherapy and Rapid Arc SIB-SBRT Treatment Plan Comparison for Carcinoma Prostate

**DOI:** 10.31557/APJCP.2020.21.4.1149

**Published:** 2020-04

**Authors:** Bijina T K, K M Ganesh, Pichandi A, Muthuselvi C A

**Affiliations:** 1 *Research and Development Center, Bharathiar University, Coimbatore, *; 2 *Department of Radiation Oncology, Healthcare Global Enterprises, Bangalore, *; 3 *Department of Radiation Physics, Kidwai Memorial Institute of Oncology, Bengaluru, India. *

**Keywords:** Prostate cancer, RapidArc, Cyberknife, Helical Tomotherapy, SIB-SBRT

## Abstract

**Background:**

This study was conducted to dosimetrically compare plan quality of Simultaneous Integrated Boost – Stereotactic Body Radiotherapy (SIB-SBRT) generated for different techniques such as Cyberknife (CK), Helical Tomotherapy (HT) and RapidArc (RA) for carcinoma prostate with same treatment margins.

**Materials and Methods:**

SIB-SBRT plans were generated for CK, HT and RA for thirteen CT data sets. The dose prescription was 45Gy in 5 fractions to GTV45 and 37.5Gy in 5 fractions to PTV37.5. The plan quality evaluation of the three techniques was done by comparing the DVH parameters, conformity index (CI) and gradient index (GI). For OAR’s mean, maximum dose and dose volumes were compared for bladder, rectum and bilateral femoral heads. The number of Monitor Units (MU) delivered and Beam-on time (BOT) were also compared.

**Results:**

D_2%_, D_50%_ and D_Mean_ to GTV45 was significantly higher in the CK compared to HT and RA (CK vs HT: p values, <0.001, 0.002 and 0.003; CK vs RA: p values, 0.001, 0.004 and 0.004) respectively. RA gives a better gradient index compared to CK and HT. Conformity indices of the RA plans were better than the CK plans (P<0.001). Normal tissue and integral dose delivered to the patient in HT and CK were found to be significantly higher than RA. The average number of MU’s and BOT were significantly higher in CK (p<0.001).

**Conclusion:**

Using the same treatment margins and dose constraints, RA achieved better target dose distribution and sparing of critical structures compared to CK and HT. RA seemed to be the optimal planning technique for SIB-SBRT treatment of carcinoma prostate.

## Introduction

Prostate cancer is one of the most common cancers in men. Various radiotherapy techniques for treating prostate cancer have been considered effective non-invasive treatment options (Kang et al., 2017). Different techniques of radiotherapy, as well as fractionation regimens, are currently used for localized prostate cancer. Among various techniques, most frequently used applied treatment modality is Intensity Modulated Radiotherapy (IMRT) among which rotational approaches such as helical tomotherapy (HT) and rapid arc (RA), can potentially deliver a higher dose per fraction, achieving high conformity and reducing the dose delivered to the surrounding organs at risk (Scobioala et al., 2019).

Promising results have also been achieved using SBRT techniques, including the linear accelerator (Linac) and CyberKnife (CK) in the treatment of prostate cancer. It has been reported that various studies describe the advantages of the above mentioned techniques for various sites including head and neck, esophageal, brain, cervical cancers, ovarian cancers, etc. (Kinhikar et al., 2014; Murthy et al., 2011). A study has reported no dosimetric advantage of CK over RA for SBRT delivery in prostate cancer (MacDougall et al., 2014). Such contrasting findings and very minimal studies regarding the optimal planning using various SBRT techniques, viz., CK, HT and RA instigated us to conduct this study comparing treatment plans of CK, HT and RA for simultaneous integrated boost (SIB) with SBRT for carcinoma prostate using same treatment plan margins for all the techniques.

## Materials and Methods

*Study Design*

A retrospective study was conducted among thirteen localized prostate cancer patients previously treated at our centre. The planning images of all the thirteen patients diagnosed with intermediate and low risk prostate cancer and treated with CK were utilized for a dosimetric comparison between CK, HT and RA. For dosimetric comparison of different SBRT techniques, treatment plans for each patient were generated. 

*Contouring*

The plain CT scan of 1.25 mm slice thickness from L5 level superiorly to mid-thigh level inferiorly was used for contouring. The entire prostate gland was contoured as CTV with 0.3cm margin given around CTV to create PTV37.5. The dominant nodule within the prostate gland was contoured as GTV45 with no margins. The same treatment margin was used for three techniques. Rectum, urinary bladder, penile bulb, small bowel and bilateral femoral heads were delineated as Organs at risks (OARs).

*Radiation therapy planning*

A SIB plan with a dose of 37.5Gy and 45Gy in 5 fractions was prescribed to PTV37.5 and GTV45 respectively. The dose constraints for all the OAR’s were set as described in Table 1 for three different techniques. The maximum dose to GTV45 was restricted to <120%. 

The RA plans were generated with True Beam STx (Varian Medical Systems Inc, Palo Alto) linac with a maximum rate of 1,400 MU/min for 6 Flattening Filter Free (FFF) beam with a dose rate of 837MU/min equipped with a high-definition multileaf collimator with 2.5-mm leaf width in the center. The treatment plans were optimized and calculated with Eclipse (Varian Medical Systems, Inc) TPS version 13.6 using an Anisotropic Analytical Algorithm (AAA). The grid sizes for optimization and dose calculation were set to 2.5 mm. Each plan consisted of two full coplanar arcs with collimator angle rotations of 30 and 330 degrees. We used Arc Geometry Tool for creation of the arcs.

HT plans were generated for the TomoH system in VoLO (Accuray, Sunnyvale, CA) TPS version 5.1.4 with a Collapsed Cone Convolution/Superposition algorithm that uses 6MV unflattened photon beam with a dose rate of 837MU/min modulated by 64 binary multileaf collimators. The plan parameters used were 2.5 mm field width, pitch value of 0.172 and modulation factor in the range 1.5 to 2. 

Cyberknife plans were generated using Multiplan planning system version 5.1.4 (Accuray, Sunnyvale, USA) using Ray tracing algorithm. Cyberknife delivers a 6 MV unflattened photon beam with a dose rate of 600 MU/min. The collimator system consists of 12 fixed cones with a size of 5 mm to 60 mm at 80 cm SAD. Double fixed collimators were chosen for planning depending on the size of the target.

*Plan evaluation parameters*

The plan quality was evaluated by comparing the dosimetric results obtained from the cumulative dose-volume histograms (DVH) of plans for all the techniques. The GTV45 and PTV37.5 were evaluated for mean doses, D_98%_, D_50%_ and D_2%_. The mean, maximum dose and the dose volumes V_80%_, V_50%_ and V_20%_, were analyzed for bladder and rectum. D_2%_ and mean dose for bilateral femoral heads were also compared. Integral dose and V_5Gy_ to body was also evaluated. Average number of Monitor Units (MU) delivered and Beam-on time (BOT) was compared among the three techniques. Conformity index (CI), dose gradient index (GI) was used to compare the efficacy of each treatment plan. The definition of each index is summarized below.

CI: The ratio used to evaluate the quality of fit of the target volume to the prescription isodose volume. It was proposed by the Radiation Therapy Oncology Group (RTOG) and modified by Paddick (2000). A smaller value of CI indicates a better conformity of the target volume.

CI _Paddick_ = TV^2^_PIV_/ (TV×V_RI_)

Where PIV, prescription isodose volume; V_RI_, volume encompassed within the reference isodose; TV, target volume.

GI: The index that represents the degree of dose drop-off outside the target volume, which was proposed by Paddick et al (2006). A smaller value of GI indicates a better degree of dose drop-off outside the target volume.

GI _Paddick_ = PIV_50_/PIV

Where PIV_50_ is the volume receiving at least 50% dose of the prescription dose; PIV is the prescription isodose volume.

*Statistical Analysis*

All the data were entered into an excel sheet and were analyzed using Statistical Package for Social Sciences (SPSS) version 18.0. The mean doses received by 2%, 50% and 98% of the GTV45 and PTV37.5 and mean doses were compared among CK, HT and RA using one-way ANOVA. The means of conformity and gradient indices, integral dose, V_5Gy_ (cc), MUs, BOT and OAR doses were also compared using one-way Analysis of Variance (ANOVA). The level of significance between the three groups was elicited using Tukey’s post hoc test which allows for multiple comparisons of CK, HT and RA. A P-value of <0.05 was considered as statistically significant.

## Results

The dose received by 2% and 50% of the GTV45 and the mean dose was significantly higher in the CK technique compared to both HT and RA (P<0.05). The dose received by 98% of GTV was significantly higher in HT compared to CK and RA (P<0.05).

**Figure 1:**
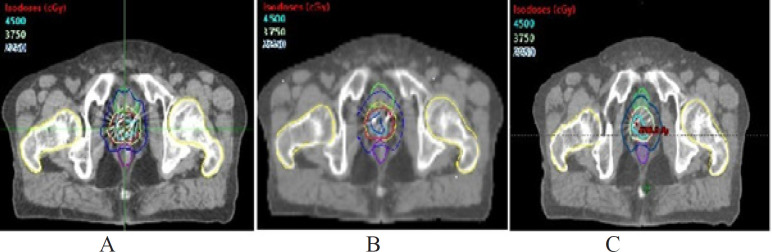
Comparison of Dose Distribution Among Three Techniques Axial Slice Showing Isodose Distribution Planned for (A) Cyberknife (B) Helical Tomotherpy and (C) Rapidarc

Similarly the dose received by 2% of the PTV37.5 was significantly higher in CK compared to both HT and RA (P<0.05) and HT delivered significantly higher dose compared to RA (P<0.05). However the dose delivery to 50%, 98% of the PTV37.5 and the mean dose did not differ in any of the techniques (Table 2). Isodose comparison of CK, HT and RA for one representative patient is shown in Figure 1.

**Figure 2:**
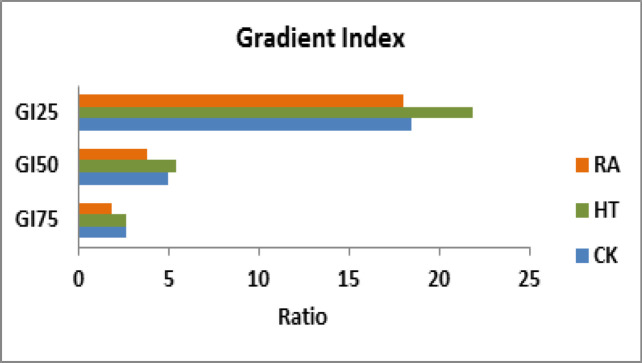
Comparison of Gradient Index, GI_75_ – Ratio of 75% Isodose Volume to Prescription Isodose Volume, GI_50_ – Ratio of 50% Isodose Volume to Prescription Isodose Volume, GI_25_ – Ratio of 25% Isodose Volume to Prescription Isodose Volume

The GI can show which of these prescription isodoses will give the steepest dose falloff outside the target, hence indicating that a dose fall off was sharper in RA compared to CK and HT (P<0.05) at 75% and 50% prescription isodose. However, at 25% prescription isodose, dose fall off was sharper in RA and CK compared to HT (P<0.05) as shown in Figure 2. The CI was less than 1 for both GTV45 and PTV37.5 among all the three techniques. The conformity index was significantly better in RA compared to both HT and CK for both GTV and PTV (P<0.05) (Table 3).

**Figure 3:**
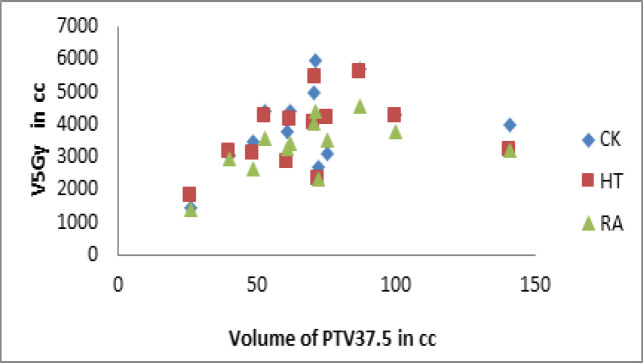
V_PTV37.5_ vs V_5Gy_; V_PTV37.5_ – Volume of PTV37.5, V_5Gy_ – Volume of Body Receiving 5Gy

The integral dose was significantly higher in CK compared to the other two treatment techniques (P<0.05). The volume of normal tissue receiving 5Gy did not differ significantly among the treatment techniques (P<0.05). Figure 3 represents the graph between volume of PTV37.5 and 5Gy volume of body. The number of monitor units and beam-on time were significantly higher in CK compared to other two techniques and it was also significantly higher in HT compared to RA (P<0.05) (Table 4).

The percentage volume of bladder receiving 80% and 50% of the dose was significantly higher in CK compared to RA and in HT compared to RA (P<0.05). However, bladder volume receiving 20% of the dose and mean dose was significantly higher in CK compared to RA and the dose received by 2% of the bladder was significantly higher in both CK and HT compared to RA (P<0.05).

The percentage volume of rectum receiving 80%, 50%, 20% of the dose, mean dose and the dose received by 2% of the rectum was significantly higher in CK compared to RA and in HT compared to RA (P<0.05). 

The mean dose to right femur was significantly higher in HT compared to CK and the dose received by 2% of the right femur was significantly higher in RA compared to CK (P<0.05). However, the mean dose and dose received by 2% of the left femur was significantly higher in CK compared to other two techniques (P<0.05) (Table 5).

**Table 1:** Dose Constraints

Structure	Volume	Constraints
GTV45	% volume receiving 45 Gy	95%
GTV37.5	% volume receiving 37.5 Gy	95%
Rectum	% receiving 37.5 Gy	<5%
	% receiving 30 Gy	<20%
	% receiving 18.75Gy	<50%
	Maximum dose to 1 cm^3^	39.5Gy
Bladder	% receiving 37.5 Gy	<5%
	% receiving 18.75 Gy	<50%
	Maximum dose to 1 cm^3^	39.5Gy
Femoral heads	Maximum point dose	30 Gy

**Table 2:** Comparison of Dose to Gross Tumor Volume (GTV45) and Planning Target Volume (PTV37.5) Among Different Treatment Techniques

DVH parameters	CK	HT	RA	(*P*-value)		
				CK vs HT	HT vs RA	RA vs CK
GTV45						
D_2%_ (Gy)	49.03 ± 0.91	47.45 ± 0.72	47.88 ± 0.39	<0.001*	0.273	0.001*
D_98%_ (Gy)	44.45 ± 0.42	45.30 ± 0.22	44.57 ± 0.21	<0.001*	<0.001*	0.592
D_50%_ (Gy)	47.18 ± 0.74	46.41 ± 0.46	46.46 ± 0.31	0.002*	0.964	0.004*
Mean Dose (Gy)	47.03 ± 0.63	46.39 ± 0.39	46.42 ±0.25	0.003*	0.993	0.004*
PTV37.5						
D_2%_ (Gy)	46.37 ± 0.92	45.19 ± 0.79	43.97 ± 0.38	0.001*	<0.001*	<0.001*
D_98%_ (Gy)	36.27 ± 0.96	36.97 ± 0.75	36.54 ± 0.37	0.051	0.290	0.637
D_50%_ (Gy)	41.04 ± 0.61	40.89 ± 0.66	40.61 ± 0.58	0.819	0.475	0.191
Mean Dose (Gy)	40.96 ± 0.49	40.87 ± 0.57	40.57 ± 0.43	0.868	0.311	0.129

*, indicates the differences to be statistically significant (P<0.05); Values represent Mean±SD; CK, Cyberknife; HT, Helical Tomotherapy; RA, Rapidarc; D_2%_, Dose to 2% volume; D_98%_, Dose to 98% volume; D_50%_, Dose to 50% volume

**Table 3:** Comparison of Dosimetric Indices Among the Three Treatment Techniques

Dosimetric indices	CK	HT	RA	(*P*-value)		
				CK vs HT	HT vs RA	RA vs CK
Gradient indices						
GI_75_	2.61 ± 0.54	2.67 ± 0.40	1.83 ± 0.22	0.918	<0.001*	<0.001*
GI_50_	4.97 ± 0.86	5.45 ± 0.82	3.78 ± 0.25	0.213	<0.001*	<0.001*
GI_25_	18.48 ± 4.51	21.81 ± 3.45	17.99 ± 1.63	0.043*	0.019*	0.935
Conformity indices						
PTV37.5	0.75 ± 0.07	0.74 ± 0.07	0.89 ± 0.01	0.863	<0.001*	<0.001*
GTV45	0.35 ± 0.11	0.49 ± 0.15	0.71 ± 0.11	0.017*	<0.001*	<0.001*

*, indicates the differences to be statistically significant (P<0.05); Values represent Mean±SD; CK, Cyberknife; HT, Helical Tomotherapy; RA, Rapidarc; GI75, Ratio of 75% of prescription dose to presctiption volume,;GI_50_, Ratio of 50% of prescription dose to presctiption volume.

**Table 4:** Comparison of other Variables Like Integral Dose, V5Gy (cc), Monitor Units (MUs) and Beam Time Among the Three Different Types of Treatment

Variables	CK	HT	RA	(*P*-value)		
				CK vs HT	HT vs RA	RA vs CK
ID (Gy-L)	86.50±18.58	57.15±14.28	50.94±16.06	<0.001*	0.603	<0.001*
V_5Gy_ (cc)	3934.62±1229.52	3715.41±1113.49	3312.33±855.14	0.863	0.610	0.316
MUs/fr	13419.68±1739.68	9565.23±1315.38	3277.77±497.94	<0.001*	<0.001*	<0.001*
BOT (min)	22.37±2.89	11.12±1.33	2.54±0.38	<0.001*	<0.001*	. <0.001*

*, indicates the differences to be statistically significant (P<0.05); Values represent Mean±SD; CK, Cyberknife; HT, Helical Tomotherapy; RA, Rapidarc; ID, Integral dose; V_5Gy_, Volume receiving 5Gy; MUs/fr, Monitor units per fraction; BOT, Beam on time

**Table 5:** Comparison of Dose Delivery to the Organs at Risk (OARs) Among the Three Different Types of Treatment

Dose to OARs	CK	HT	RA	(P-value)		
				CK vs HT	HT vs RA	RA vs CK
Bladder						
V _80%_ (%)	1.45 ± 0.89	1.39 ± 1.10	0.33 ± 0.38	0.982	0.008*	0.005*
V _50%_ (%)	14.82 ± 6.74	13.71 ± 5.89	6.88 ± 2.73	0.859	0.008*	0.002*
V _20%_ (%)	51.87 ± 14.55	47.51 ± 12.15	38.24 ± 7.42	0.616	0.124	0.015*
D_Mean_ (Gy)	12.63 ± 1.91	11.12 ± 2.83	8.63 ± 1.41	0.181	0.014	<0.001*
D_2%_ (Gy)	34.82 ± 2.17	34.14 ± 2.96	28.75 ± 2.88	0.799	<0.001*	<0.001*
Rectum						
V _80%_ (%)	1.56 ± 1.07	1.96 ± 1.32	0.27 ± 0.23	0.563	<0.001*	0.005*
V _50%_ (%)	16.60 ± 4.52	17.66 ± 4.75	6.96 ± 2.26	0.779	<0.001*	<0.001*
V _20%_ (%)	59.49 ± 8.40	58.59 ± 9.89	45.76 ± 9.71	0.967	0.004*	0.002*
D_Mean_ (Gy)	13.28 ± 1.66	12.52 ± 2.61	9.59 ± 1.35	0.586	0.001*	<0.001*
D_2%_ (Gy)	34.67 ± 1.49	35.51 ± 1.86	29.13 ± 2.53	0.543	<0.001*	<0.001*
Right Femur						
D_Mean_ (Gy)	7.15 ± 1.42	8.92 ± 1.57	8.72 ± 2.06	0.114	0.834	0.033*
D_2%_ (Gy)	11.16 ± 1.39	12.62 ± 1.97	13.03 ± 2.01	0.031	0.949	0.063
Left Femur						
D_Mean_ (Gy)	5.42 ± 0.99	8.29 ± 1.55	8.79 ± 2.04	0.016	0.982	0.010*
D_2%_ (Gy)	10.64 ± 1.44	12.34 ± 1.69	12.44 ± 1.29	<0.001*	0.707	<0.001*

*, indicates the differences to be statistically significant (P<0.05); V80%, Volume receiving 80% of prescription dose; V_50%_, Volume receiving 50% of prescription dose; V_20%_, Volume receiving 20% of prescription dose, D_Mean_, Mean dose, D_2%_, Dose to 2% volume

## Discussion

With the conflicting results in the literature with respect to different SBRT treatment techniques like CK, different types of intensity-modulated radiation therapy (IMRT) for cancer prostate, this current study was conducted and we have found some interesting results which would be discussed as below. To deliver a high dose to the prostate, different radiation modalities exist like HT, RA and CK with improved dose delivery techniques enabling better coverage of a high dose region and safe delivery of high total doses to the planning treatment volume (PTV) with sparing of organs at risk and adjacent healthy tissue (Slosarek et al., 2015).

Seppala et al., (2017) found that the mean dose was significantly higher in the prostate and PTV with CK. Similarly, in our study, the mean dose to the GTV45 was significantly higher in the CK technique compared to other two and it did not differ for the PTV.

Hegazy et al., (2016) found that all dose constraints regarding PTV coverage were similarly achieved by both plans generated by the RA and CK except for the maximal doses generated by the RA plans which were statistically significant lower than those of the CK plans. Tree et al., (2013) have shown that RA and CK can produce clinically acceptable plans. Macdougall et al., (2014) showed no distinct dosimetric advantage to choose CK over RA and also found that CK failed to achieve the desired PTV homogeneity constraint in two cases. Chen et al., (2017) have recorded tomotherapy plans to be better in terms of better dose homogeneity and target coverage compared to CK plans.

Lin et al.,(2014) found that conformity and heterogeneity indices of the RA plans were better than the CK plans similar to current study. The CI in our study was significantly better in RA compared to both HT and CK for both GTV45 and PTV37.5.

Slosarek et al., (2015) have found the integral dose delivered to the patient in HT and CK was found to be significantly higher than in RA/VMAT similar to the current study wherein, the integral dose was significantly higher in CK compared to the other two treatment techniques. Seppala J et al., (2017) also have found it to be significantly higher in cyberknife similar to current study, though they have not mentioned regarding the significance among other techniques. However, in our study, the number of MUs and BOT were significantly higher in HT compared to RA.

MacDougall et al., (2014) noted at doses <35 Gy, normal tissues received higher doses with CK and is in line with our findings wherein bladder and rectum dose was higher in CK and the comparison in their study, was only between CK and RA. Seppala et al., (2017) also found that it was highest in CK, similar to our study and they have linked it to the non-coplanar nature of the delivery system and also because of the system’s mechanical inability to irradiate directly from lateral oblique and posterior directions. Chen et al., (2017) have concluded saying that HT might have advantages over CK, mainly in rectal sparing. The mean maximum dose to right femur was significantly higher in RA compared to CK and left femur in CK. The dose to femur is in line with Seppala et al., (2017).

Though the study has shown dosimetrically comparable results with respect to RA and HT and also few advantages over cyber knife, it remains a query in clinical practice.It needs to be evaluated with respect to delivery accuracy or intra-fraction target motion and positioning, which is better addressed with CK, equipped with orthogonal X-rays to track the gold fiducials inserted in the prostate gland in real time, linac mounted on robotic arm. Moreover, this cannot be done in RA or HT without stopping the treatment in-between, although can deliver the treatment in very short duration. Each technique also uses diverse planning systems, optimization criteria and algorithms which cannot be standardized. The high-dose regions within the prostate were significantly higher with CK than others. Hence might implicate positive effect on the tumor control probability clinically which needs an evaluation again. The reason for RA and HT producing better GI could be because of treatment delivery posteriorly to the patient, which is limited with CK. In addition to this, the MLCs align to fit in the target which helps in better plan when compared to the fixed collimator size of CK. 

In conclusion, there is a significant dosimetric advantage of RA over other two techniques. Using the same treatment margins and dose constraints, RA achieved better target dose distribution and sparing of critical structures compared to CK and HT with minimal treatment delivery time. RA seemed to be the optimal planning technique for SIB-SBRT treatment of carcinoma prostate.

## Conflict of interest

Authors have declared no conflict of interest.
